# Supplementary material for: Analysis of PPARγ Signaling Activity in Psoriasis
Source: Int J Mol Sci. 2021 Aug 10;22(16):8603. doi: 10.3390/ijms22168603 (PMC8395241; doi:10.3390/ijms22168603)
Supplement: Supplementary file 1 [file ijms-22-08603-s001.zip › Supplemental materials_Analysis of PPARg signaling activity in psoriasis/Pathway models/Models images and html files/Differentiation of psoriatic T cells/7866.html]

MYD88


# Protein MYD88

|  |  |
| --- | --- |
| URN | urn:agi-llid:4615 |
| Total Entities | 0 |
| Connectivity | 3523 |
| Name | MYD88 |
| Description | myeloid differentiation primary response 88 |
| Notes | This gene encodes a cytosolic adapter protein that plays a central role in the innate and adaptive immune response. This protein functions as an essential signal transducer in the interleukin-1 and Toll-like receptor signaling pathways. These pathways regulate that activation of numerous proinflammatory genes. The encoded protein consists of an N-terminal death domain and a C-terminal Toll-interleukin1 receptor domain. Patients with defects in this gene have an increased susceptibility to pyogenic bacterial infections. Alternate splicing results in multiple transcript variants. [provided by RefSeq, Feb 2010] |
| Primary Cell Localization | Cytoplasm |
| Class | Receptor |
| ObjectType | Protein |

---

|  |  |
| --- | --- |
| Pathway | Osteoarthritis |
|  | Fatty Acids Block TLR Signaling in Enteroendocrine Cell |
|  | I-cell: CCK-8 Secretion and Regulation of Eating Behavior |
|  | Toll-like Receptors Act through MYD88-TIRAP Signaling |
|  | Antiviral Signaling through Pattern Recognition Receptors |
|  | Toll-like Receptors Act through MYD88 Signaling |
|  | TLR4 Signaling in Leukocytes |
|  | Dectin-2 (CLEC6A)/Mincle (CLEC4E)/BDCA2 (CLEC4C) Signaling |
|  | Th1-Cell Differentiation |
|  | Th17-Cell Differentiation |
|  | Toll-like Receptors in Sterile Inflammation |
|  | Toll-like Receptor Independent Sterile Inflammation |
|  | Basophil Activation |
|  | Eosinophil Survival by Cytokine Signaling |
|  | Mast-Cell Activation without Degranulation |
|  | Mast-Cell Activation without Degranulation through IL33/IL1RL1 Signaling |
|  | Mast-Cell Activation without Degranulation through IL1R1 and TLR Signaling |
|  | Macrophage M1 Lineage |
|  | CD16/CD14 Proinflammatory Monocyte Activation |
|  | AHR Signaling in M1 Macrophages Function |
|  | anti-Inflammatory Function of Macrophage M2 Lineage |
|  | AXL Receptor Inhibits Macrophages and Dendritic Cells Function |
|  | Neutrophil Recruitment and Priming |
|  | Negative Acute Phase Proteins Synthesis |
|  | Positive Acute Phase Proteins Synthesis |
|  | Vascular Endothelial Cell Activation by Cytokines |
|  | Vascular Endothelial Cell Activation by Blood Coagulation Factors |
|  | Adipokines Production by Adipocyte |
|  | IL1B Expression Targets -> Nociception |
|  | Proteins Involved in Atherosclerosis |
|  | Dendritic Cells Function in Atherosclerosis |
|  | RAGE/AGER and S100 Proteins in Cardiovascular Injury |
|  | Low-Density Lipoproteins and Chemokines in Atherosclerosis |
|  | Th1-Cells Activation and Proliferation in Atherosclerosis |
|  | Myocardial Infarction |
|  | Lymphocyte Mediated Myocardial Injury in Myocarditis |
|  | Proteins Involved in Myocarditis |
|  | Toll-like Receptors (TLR) in Antiviral Innate Immune Response |
|  | Inflammatory Reaction in Acne Vulgaris |
|  | Keratinocyte Proliferation in Acne Vulgaris |
|  | Mast-Cells Activation in Atopic Dermatitis |
|  | Proteins Involved in Atopic Dermatitis |
|  | T-Cells Differentiation Block in Psoriasis |
|  | Dendritic Cells Function in Psoriasis |
|  | Apoptotic Keratinocytes Clearance Recession in Systemic Lupus Erythematosus |
|  | Dendritic Cell Activation in Systemic Lupus Erythematosis |
|  | Th17-Cell Function in Systemic Lupus Erythematosus |
|  | Neutrophil and Macrophage Function in Systemic Lupus Erythematosus |
|  | Vitiligo |
|  | T-Cell Cytotoxic Response against Melanocytes in Vitiligo |
|  | Plasmacytoid Dendritic Cell in Diabetes Mellitus Type 1 |
|  | TLR2 Signaling in Treg-Cell in Type 1 Diabetes (Animal Model) |
|  | Toll-like Receptors in beta-Cell |
|  | TLR7 and TLR9 in Plasmacytoid Dendritic Cell |
|  | Conventional Dendritic Cell Role in Diabetes Mellitus Type 1 |
|  | Insulin Resistance in Myocytes Induced by Obesity |
|  | Hepatic Stellate Cells in non-Alcoholic Fatty Liver Disease |
|  | Proteins Involved in non-Alcoholic Fatty Liver Disease |
|  | beta-Cell Destruction through Cytokines in Diabetes Mellitus |
|  | beta-Cell Destruction in Diabetes Mellitus Type 1 |
|  | Adipokines Production by Adipocyte Impaired in Obesity |
|  | CD4+T-Cell Response in Celiac Disease |
|  | Celiac Disease |
|  | Crohn's Disease |
|  | Dendritic Cell Dysfunction in Crohn's disease |
|  | Dendritic Cell Function in Ulcerative Colitis |
|  | Epithelial Cell in the Innate Immune Response in Ulcerative Colitis |
|  | Leukocyte Migration toward the Endothelial Cell in Ulcerative Colitis |
|  | Nicotine anti-Inflammatory Effect in Ulcerative Colitis |
|  | Antiphospholipid Antibodies in Endothelial Cell |
|  | B-Cell Chronic Lymphocytic Leukemia |
|  | Diffuse Large-B-Cell Lymphoma |
|  | Diffuse Large-B-Cell Lymphoma ABC Subtype |
|  | Proteins Involved in Hodgkin Lymphoma |
|  | Mucosa-Associated Lymphoid Tissue (MALT) Lymphoma |
|  | Splenic Marginal Zone Lymphoma |
|  | Antiphospholipid Antibodies and Endothelial Cell Interaction |
|  | IL33 Signaling Related Eosinophilia in Asthma |
|  | TSLP Signaling in Bronchial Epithelial Cell in Asthma |
|  | Club-Cell Role in Asthma |
|  | Basophil Activation in Asthma |
|  | Antigen-Presenting Cell Role in Asthma |
|  | Th17-Cell Differentiation in Asthma |
|  | Mucin Production in Goblet Airway Epithelial Cells |
|  | CFTR Facilitates Oxidative Stress in Airway Epithelium in CF |
|  | Airway Epithelial Cell Dysfunction |
|  | CFTR Expression in Epithelial Cells (Class I Mutations) |
|  | CFTR Related Ion-Channel Dysfunction in Cystic Fibrosis Airway Epithelium (Class III Mutations) |
|  | Osteoclast Activation in Gout |
|  | Neutrophil Recruitment in Sinovium in Gout |
|  | Monocytes Function in Gout |
|  | TNF and IL1B Induce Metalloproteinase Synthesis in Osteoarthritis |
|  | IL1B Induced Arthralgia |
|  | Dendritic Cell Dysfunction in Psoriatic Arthritis |
|  | Th17-Cell and Th1 Immune Responsein Psoriatic Arthritis |
|  | Osteoclast Activation in Psoriatic Arthritis |
|  | Synovial Fibroblast Activation in Psoriatic Arthritis |
|  | Osteoclast Activation in Rheumatoid Arthritis |
|  | Synovial Fibroblast Activation by Citokines in Rheumatoid Arthritis |
|  | TLR2 Induced Synovial Fibroblast Activation in Rheumatoid Arthritis |
|  | Autocrine Cytokine/Chemokine Loops in Systemic Scleroderma |
|  | Th17-Cell Numbers Reduction in HIV |
|  | Toll-like Receptors (TLR) in Periodontitis |
|  | IL1 Receptor Antagonist (IL1RN) Deficiency (DIRA) |
|  | PAPA Syndrome |
|  | TNF Receptor Associated Periodic Syndrome |
|  | Mevalonate Kinase Deficiency |
|  | Blau Syndrome |
|  | IL36 Receptor Antagonist (IL1F5) Deficiency (DITRA) |
|  | Cryopyrin (NLPR3) Associated Periodic Syndromes |
|  | Familial Cold Autoinflammatory Syndrome 2 |
|  | Familial Mediterranean Fever |
|  | Majeed Syndrome |
|  | Gluten Impact on Neuronal System (Hypothesis) |
|  | Microglia Activation in Alzheimer's Disease |
|  | HMGB1 and IL1B in Neuroinflammation |
|  | HMGB1 and IL1B in Neuronal Hyperexcitation |
|  | Microglia Activation in Parkinson's Disease |
|  | Proteins Involved in Amyotrophic Lateral Sclerosis |
|  | Subepithelial Fibroblasts in Middle Ear Cholesteatoma |
|  | Cholesteatoma |
|  | HPV E6 and E7 Induced Disruption of TNFR/NF-kB Signaling in Keratinocytes |
|  | Proteins Involved in Otitis Media |
|  | Cytokines Trigger Otitis Media |
|  | MUC5AC Expression by NTHi and S. Pneumoniae |
|  | Immune System Role in Otitis Media |
|  | Alveolar Macrophage Negative Regulation Declined in COPD |
|  | Alveolar Macrophages Dysfunction in COPD |
|  | T-Cell Cytotoxic Activation |
|  | Alveolar Epithelial Cell Dysfunction in COPD |
|  | Mucin Hyperproduction in Goblet and Mucous Cells |
|  | Endothelial Cell Dysfunction in Glomerulonephritis |
|  | Toll-like Receptors in Pyelonephritis |
|  | Endothelial Cell Dysfunction in Pyelonephritis |
|  | Proteins with Altered Expression in Cancer-Associated Sustaining of Proliferative Signaling |
|  | Genes with Mutations in Cancer-Associated Sustaining of Proliferative Signaling |
|  | NFKB Canonical Signaling Activation in Cancer |
|  | STAT3 and NFkB Activate Inflammation-Induced Tumorigenesis |
|  | NFKB Signaling Activation by Blocking of Tumor Suppressors |
|  | IDO1 in Cancer Immune Escape |
|  | IL1R -> STAT3 Signaling |
|  | IL1R -> NF-kB Signaling |
|  | IL21 Expression Targets |
|  | Immunoglobulin Superfamily -> Expression Targets in Lymphoid System and Blood |
|  | C5AR1 -> Expression Targets in Lymphoid System and Blood |
|  | NF-kB Canonical Signaling |
|  | TLR4 -> AP-1 Expression Targets |
|  | TLR5 Expression Targets |
|  | TLR7 Expression Targets |
|  | TLR4 -> AP-1/EGR1/HIF1A Expression Targets |
|  | TLR1 -> 2/6 Expression Targets |
|  | IL1B Expression Targets |
|  | TLR4 -> NF-kB/IRF Expression Targets |
|  | IL1A Expression Targets |
|  | TLR9 Expression Targets |
|  | TLR4 -> IRF Signaling |
|  | TLR4/5/7/9 -> NF-kB Signaling |
|  | TLR1/2/6 -> NF-kB Signaling |
|  | TLR -> AP-1 Signaling |
|  | pathway 4 |
|  | PPAR Psoriasis |
|  | Model of PPARG signaling in psoriasis |
|  | PPARG negative regulators and targets |
|  | Model of PPARG related pathways in psoriasis (short version) |
|  | sars-cov no binding |
|  | dsRNA induces innate immunity and INFs in alveolar cells |
|  | Innate immunity in Leukocytes |
|  | AGER feed forward loop in innate immunity |
|  | Dendritic Cell Activation in COVID19 |
|  | 1\_Inflammation in keratinocytes in the pathology of acne |
|  | 2\_Acute phase initiation in atopic dermatitis |
|  | 1\_Differentiation of psoriatic T cells |
|  | 1\_TNF and IL1B provoke ECM degradation in osteoarthritis |
|  | 2\_Chondrocyte death in osteoarthritis |
|  | 1\_The role of Toll-like receptors in pyelonephritis |
|  | 2\_The role of endothelial cells in pyelonephritis |
|  | 1\_Insufficient activation of immune response in the middle ear epithelium cells in otitis media |
|  | 2\_Pathogens stimulate mucins expression in the middle ear |
|  | 1\_Low-density lipoprotein internalization and the accumulation of lipids in atherosclerosis |
|  | 3\_Arterial wall inflammation and calcification in atherosclerosis |
|  | 1\_Cigarette smoke causes dysfunction and production of inflammatory mediators in alveolar epithelial cells |
|  | 3\_Role of alveolar macrophages in pulmonary tissue destruction in COPD |
|  | 1\_Th2 cell response in asthma |
|  | 3\_1\_Mucus accumulation in asthma |
|  | 2\_2\_Eosinophilia and neutrophilia in asthma: eosinophil apoptosis |
|  | 4\_Mucin hyperproduction in goblet and mucous cells in COPD |
|  | 1\_Defects in response to pathogens in the gut promotes inflammation in Crohn's disease |
|  | 3\_Leukocyte migration toward the endothelial cells in the intestine microvasculature |
|  | 2\_Intestine epithelial cells drive immunological responses in ulcerative colitis |
|  | 1\_Intestine epithelial cell dysfunction in ulcerative colitis |
|  | 4\_1\_Polymorphisms associated with inflammatory bowel diseases |
|  | 2\_Lipotoxicity in NAFLD |
|  | 1\_CD4+ T cell and epithelial cell response in celiac disease |
|  | 5\_Human papilloma virus E6 and E7 proteins promote epithelial cell proliferation |
|  | 4\_3\_Insulin resistance: FFA-related insulin resistance |
|  | 1\_CFTR expression and degradation in epithelial cells in cystic fibrosis (class I-II mutations) |
|  | 1\_Antigen-presenting cells promote islet dysfunction and immune system activation in T1D |
|  | 3\_Islet beta-cell destruction in T1D |
|  | 2\_Neuron and Schwann cell death |
|  | Figure 4\_Diabetes mellitus Type 1: conventional dendritic cell |
|  | drug-target pairs direct regulation |
|  | Model of PPARG signaling in psoriais (tested) |
|  | mapk1 direct regulators |
|  | Differentiation of psoriatic T cells |

---

|  |  |
| --- | --- |
| Group | Genes with Mutations Associated with Atopic Dermatitis |
|  | Genes with Mutations Associated with Splenic Marginal Zone Lymphoma |
|  | other partners for SARS |

---

|  |  |
| --- | --- |
| MedScan ID | 4615 |

---

|  |  |
| --- | --- |
| LocusLink ID | 4615 |
|  | 17874 |
|  | 301059 |
|  | 29638 |

---

|  |  |
| --- | --- |
| Alias | OTTHUMP00000209060 |
|  | myeloid differentiation primary response gene (88) |
|  | MYD88 |
|  | MYD88, innate immune signal transduction adaptor |
|  | myeloid differentiation factor 88 |
|  | Myeloid differentiation primary response protein MyD88 |
|  | Myeloid differentiating primary response protein MyD88 |
|  | OTTMUSP00000018013 |
|  | myeloid differentiation protein 88 |
|  | myeloid differentiation marker 88 |
|  | OTTHUMP00000208595 |
|  | myeloid differentiating primary response gene (88) |
|  | MYD88D |
|  | mutant myeloid differentiation primary response 88 |
|  | myeloid differentiation primary response 88 |
|  | myeloid differentiation primary response protein 88 |
|  | myeloid differentiation primary response gene 88 |

---

|  |  |
| --- | --- |
| GO ID | 0070976 |
|  | 0035325 |
|  | 0005123 |
|  | 0043621 |
|  | 0070935 |
|  | 0002755 |
|  | 0008063 |
|  | 0006915 |
|  | 0007166 |
|  | 0071260 |
|  | 0140052 |
|  | 0050830 |
|  | 0042742 |
|  | 0006954 |
|  | 0045087 |
|  | 0070498 |
|  | 0043066 |
|  | 0006909 |
|  | 0043123 |
|  | 0051092 |
|  | 1900017 |
|  | 0010628 |
|  | 0032740 |
|  | 0032747 |
|  | 0032755 |
|  | 0032757 |
|  | 0032481 |
|  | 0050727 |
|  | 0070555 |
|  | 0007165 |
|  | 0034162 |
|  | 0002224 |
|  | 0060337 |
|  | 0005737 |
|  | 0005829 |
|  | 0010008 |
|  | 0005634 |
|  | 0005886 |
|  | 0005121 |
|  | 0005149 |
|  | 0005102 |
|  | 0007254 |
|  | 0019221 |
|  | 0090557 |
|  | 0006955 |
|  | 0002376 |
|  | 0016064 |
|  | 0009682 |
|  | 0031663 |
|  | 0044130 |
|  | 0046330 |
|  | 0045080 |
|  | 0050671 |
|  | 0048661 |
|  | 0032760 |
|  | 0042127 |
|  | 2000338 |
|  | 2000341 |
|  | 0010468 |
|  | 0032675 |
|  | 1902622 |
|  | 0032680 |
|  | 0032496 |
|  | 0002238 |
|  | 0032494 |
|  | 0009615 |
|  | 0007178 |
|  | 0045351 |
|  | 0014069 |
|  | 0032991 |
|  | 0005622 |
|  | 0034146 |
|  | 0006916 |
|  | 0034142 |
|  | 0005515 |
|  | 0034134 |
|  | 0034130 |
|  | 0043234 |
|  | 0048011 |
|  | 0034166 |
|  | 0038123 |
|  | 0045944 |
|  | 0038124 |
|  | 0042802 |
|  | 0004888 |
|  | 0031224 |
|  | 0007249 |
|  | 0004871 |
|  | 0016020 |
|  | 0042535 |

---

|  |  |
| --- | --- |
| KEGG ID | hsa:4615 |
|  | mmu:17874 |
|  | rno:301059 |

---

|  |  |
| --- | --- |
| MedLine Reference | 10383454 |
|  | 10854325 |
|  | 10880445 |
|  | 10952994 |
|  | 11544529 |
|  | 11743586 |
|  | 11828002 |
|  | 11854210 |
|  | 11880380 |
|  | 11909531 |
|  | 11960013 |
|  | 12391239 |
|  | 12477932 |
|  | 12531807 |
|  | 12646618 |
|  | 12659850 |
|  | 12724322 |
|  | 12761501 |
|  | 12860405 |
|  | 12865424 |
|  | 12885415 |
|  | 14573621 |
|  | 14630816 |
|  | 14702039 |
|  | 15004556 |
|  | 15342556 |
|  | 15361868 |
|  | 15489334 |
|  | 15623538 |
|  | 15665823 |
|  | 15728506 |
|  | 15760459 |
|  | 15845500 |
|  | 15849357 |
|  | 16189514 |
|  | 16203735 |
|  | 16236719 |
|  | 16286016 |
|  | 16293622 |
|  | 16365431 |
|  | 16424162 |
|  | 16482509 |
|  | 16517734 |
|  | 16517750 |
|  | 16644730 |
|  | 17363736 |
|  | 17507480 |
|  | 17513785 |
|  | 8957090 |
|  | 9013863 |
|  | 9344657 |
|  | 9374458 |
|  | 9716450 |
|  | 10047546 |
|  | 10349636 |
|  | 10435584 |
|  | 10465784 |
|  | 10607756 |
|  | 10922068 |
|  | 11025209 |
|  | 11042159 |
|  | 11067888 |
|  | 11076861 |
|  | 11217851 |
|  | 11286707 |
|  | 11313410 |
|  | 11500829 |
|  | 11521059 |
|  | 11526399 |
|  | 11698470 |
|  | 11739528 |
|  | 11823477 |
|  | 11911827 |
|  | 11923871 |
|  | 12055206 |
|  | 12218091 |
|  | 12244183 |
|  | 12244184 |
|  | 12244198 |
|  | 12270723 |
|  | 12447442 |
|  | 12466851 |
|  | 12538665 |
|  | 12559494 |
|  | 12626561 |
|  | 12682257 |
|  | 12695520 |
|  | 12714520 |
|  | 12719478 |
|  | 12750407 |
|  | 12819136 |
|  | 12847214 |
|  | 12855817 |
|  | 12872135 |
|  | 12874236 |
|  | 12902324 |
|  | 12917260 |
|  | 12928382 |
|  | 12933875 |
|  | 12960345 |
|  | 14517275 |
|  | 14530329 |
|  | 14563635 |
|  | 14568952 |
|  | 14579265 |
|  | 14625548 |
|  | 14634130 |
|  | 14636418 |
|  | 14638820 |
|  | 14638995 |
|  | 14668429 |
|  | 14670305 |
|  | 14698224 |
|  | 14716310 |
|  | 14734753 |
|  | 14742526 |
|  | 14742577 |
|  | 14744795 |
|  | 14764662 |
|  | 14768061 |
|  | 14971048 |
|  | 14976261 |
|  | 14993594 |
|  | 15004134 |
|  | 15004140 |
|  | 15034058 |
|  | 15034062 |
|  | 15034070 |
|  | 15034168 |
|  | 15034566 |
|  | 15039335 |
|  | 15039368 |
|  | 15067049 |
|  | 15128790 |
|  | 15128822 |
|  | 15128825 |
|  | 15137944 |
|  | 15153511 |
|  | 15155621 |
|  | 15190057 |
|  | 15197227 |
|  | 15214027 |
|  | 15240744 |
|  | 15249654 |
|  | 15258598 |
|  | 15260992 |
|  | 15265925 |
|  | 15265935 |
|  | 15302901 |
|  | 15307830 |
|  | 15322035 |
|  | 15322195 |
|  | 15331330 |
|  | 15345224 |
|  | 15353553 |
|  | 15368304 |
|  | 15375173 |
|  | 15452235 |
|  | 15492225 |
|  | 15507117 |
|  | 15517610 |
|  | 15534227 |
|  | 15539158 |
|  | 15542452 |
|  | 15593126 |
|  | 15599404 |
|  | 15606799 |
|  | 15611251 |
|  | 15615857 |
|  | 15618166 |
|  | 15630134 |
|  | 15634894 |
|  | 15650773 |
|  | 15650774 |
|  | 15654326 |
|  | 15654341 |
|  | 15661931 |
|  | 15671286 |
|  | 15694359 |
|  | 15709004 |
|  | 15709016 |
|  | 15711573 |
|  | 15714580 |
|  | 15723296 |
|  | 15724245 |
|  | 15731053 |
|  | 15731095 |
|  | 15765149 |
|  | 15778492 |
|  | 15781585 |
|  | 15782199 |
|  | 15800576 |
|  | 15814710 |
|  | 15814731 |
|  | 15815647 |
|  | 15826931 |
|  | 15845454 |
|  | 15855642 |
|  | 15890912 |
|  | 15895089 |
|  | 15905587 |
|  | 15922717 |
|  | 15944297 |
|  | 15956283 |
|  | 15964841 |
|  | 15972526 |
|  | 15972679 |
|  | 16002681 |
|  | 16002682 |
|  | 16002720 |
|  | 16025156 |
|  | 16039576 |
|  | 16040976 |
|  | 16041030 |
|  | 16041408 |
|  | 16047338 |
|  | 16052598 |
|  | 16081822 |
|  | 16116197 |
|  | 16116224 |
|  | 16141072 |
|  | 16141073 |
|  | 16142239 |
|  | 16148103 |
|  | 16148139 |
|  | 16148144 |
|  | 16204620 |
|  | 16207247 |
|  | 16210631 |
|  | 16216974 |
|  | 16226506 |
|  | 16236128 |
|  | 16237045 |
|  | 16237098 |
|  | 16237099 |
|  | 16239556 |
|  | 16244651 |
|  | 16254214 |
|  | 16260486 |
|  | 16272281 |
|  | 16272328 |
|  | 16272344 |
|  | 16275884 |
|  | 16276483 |
|  | 16292312 |
|  | 16301743 |
|  | 16323245 |
|  | 16339557 |
|  | 16365150 |
|  | 16369011 |
|  | 16401773 |
|  | 16407889 |
|  | 16413925 |
|  | 16413926 |
|  | 16413927 |
|  | 16424940 |
|  | 16428431 |
|  | 16428731 |
|  | 16440059 |
|  | 16456012 |
|  | 16456013 |
|  | 16461338 |
|  | 16461893 |
|  | 16473828 |
|  | 16473830 |
|  | 16477040 |
|  | 16491077 |
|  | 16495516 |
|  | 16513388 |
|  | 16518342 |
|  | 16552045 |
|  | 16552075 |
|  | 16554296 |
|  | 16574902 |
|  | 16609927 |
|  | 16648192 |
|  | 16670325 |
|  | 16670327 |
|  | 16703513 |
|  | 16703567 |
|  | 16709840 |
|  | 16710369 |
|  | 16714560 |
|  | 16714586 |
|  | 16714594 |
|  | 16732281 |
|  | 16777969 |
|  | 16782034 |
|  | 16782035 |
|  | 16785556 |
|  | 16829620 |
|  | 16832055 |
|  | 16874736 |
|  | 16878177 |
|  | 16879997 |
|  | 16886064 |
|  | 16888020 |
|  | 16892068 |
|  | 16893899 |
|  | 16921381 |
|  | 16926427 |
|  | 16935388 |
|  | 16951362 |
|  | 16954198 |
|  | 16955143 |
|  | 16980981 |
|  | 16983331 |
|  | 16990136 |
|  | 17000122 |
|  | 17015716 |
|  | 17018642 |
|  | 17027299 |
|  | 17030581 |
|  | 17034585 |
|  | 17038589 |
|  | 17053835 |
|  | 17055754 |
|  | 17057095 |
|  | 17060475 |
|  | 17074859 |
|  | 17085599 |
|  | 17118979 |
|  | 17135446 |
|  | 17159982 |
|  | 17167049 |
|  | 17178920 |
|  | 17178921 |
|  | 17185603 |
|  | 17190817 |
|  | 17194808 |
|  | 17200722 |
|  | 17202381 |
|  | 17267571 |
|  | 17275788 |
|  | 17283089 |
|  | 17289654 |
|  | 17292937 |
|  | 17329566 |
|  | 17339355 |
|  | 17353199 |
|  | 17353291 |
|  | 17353783 |
|  | 17357057 |
|  | 17361131 |
|  | 17372011 |
|  | 17389381 |
|  | 17398124 |
|  | 17404295 |
|  | 17417641 |
|  | 17433728 |
|  | 17442966 |
|  | 17450126 |
|  | 17450144 |
|  | 17452470 |
|  | 17456769 |
|  | 17458858 |
|  | 17469144 |
|  | 17475853 |
|  | 17486093 |
|  | 17496895 |
|  | 17567694 |
|  | 17573354 |
|  | 17576821 |
|  | 17579078 |
|  | 17591966 |
|  | 17615358 |
|  | 17615359 |
|  | 17617634 |
|  | 17638914 |
|  | 17658280 |
|  | 17664289 |
|  | 17668900 |
|  | 17705131 |
|  | 17707128 |
|  | 17723216 |
|  | 2374694 |
|  | 7567467 |
|  | 9575168 |
|  | 9697844 |
|  | 12616494 |
|  | 14962484 |
|  | 15890643 |
|  | 16199478 |
|  | 16259957 |
|  | 16954392 |

---

|  |  |
| --- | --- |
| Organism | Homo sapiens {Organism urn:agi-taxid:9606} |
|  | Mus musculus {Organism urn:agi-taxid:10090} |
|  | Rattus norvegicus {Organism urn:agi-taxid:10116} |
|  | Homo sapiens |
|  | Mus musculus |
|  | Rattus norvegicus |

---

|  |  |
| --- | --- |
| Mouse chromosome position | 9 71.33 cM |
|  | 9 70.0 cM |

---

|  |  |
| --- | --- |
| OMIM ID | 602170 |
|  | 612260 |
|  | 153600 |

---

|  |  |
| --- | --- |
| Rat chromosome position | 8q32 |

---

|  |  |
| --- | --- |
| Hugo ID | 7562 |
|  | HGNC:7562 |

---

|  |  |
| --- | --- |
| Human chromosome position | 3p22.2 |
|  | 3p22 |

---

|  |  |
| --- | --- |
| Swiss-Prot Accession | Q99836.1 |
|  | P22366 |
|  | Q3U7M4 |
|  | P22366.3 |
|  | Q6Y1S1 |
|  | Q6Y1S1.2 |
|  | Q99836 |
|  | B4DKH8 |
|  | B4DKU4 |
|  | B4DQ60 |
|  | B4DQ72 |
|  | J3KPU4 |
|  | J3KQ87 |
|  | J3KQJ6 |
|  | P78397 |
|  | Q53XS7 |
|  | O35916 |
|  | Q4QRC0 |
|  | A0A0A0MSI9 |
|  | A0A0A0MS70 |
|  | A0A0A0MST0 |
|  | Q0PM15 |
|  | Q0PM16 |
|  | Q3TIB3 |
|  | Q3U6W5 |
|  | Q3UDG9 |
|  | Q8C1X0 |

---

|  |  |
| --- | --- |
| PIR ID | S11226 |

---

|  |  |
| --- | --- |
| GenBank ID | NC\_000003 |
|  | NM\_001172567 |
|  | NP\_001166038 |
|  | NM\_002468 |
|  | NP\_002459 |
|  | NM\_001365876 |
|  | NP\_001352805 |
|  | NM\_001172569 |
|  | NP\_001166040 |
|  | NM\_001172568 |
|  | NP\_001166039 |
|  | NM\_001365877 |
|  | NP\_001352806 |
|  | NM\_001172566 |
|  | NP\_001166037 |
|  | NG\_016964 |
|  | AP006309 |
|  | CH471055 |
|  | EAW64520 |
|  | EAW64521 |
|  | KU886048 |
|  | ANN25047 |
|  | KX098585 |
|  | ANK58152 |
|  | KX138263 |
|  | ANK58154 |
|  | KX138264 |
|  | ANK58155 |
|  | KX138265 |
|  | ANK58156 |
|  | AB446470 |
|  | BAG55247 |
|  | AK097983 |
|  | AK124685 |
|  | AK296570 |
|  | BAG59190 |
|  | AK296716 |
|  | BAG59306 |
|  | AK298650 |
|  | BAG60822 |
|  | AK298666 |
|  | BAG60834 |
|  | AK300051 |
|  | BAG61860 |
|  | AK300448 |
|  | BAG62170 |
|  | AK304676 |
|  | BAG65448 |
|  | AL832906 |
|  | BC013589 |
|  | AAH13589 |
|  | BP242808 |
|  | BP339466 |
|  | BT007376 |
|  | AAP36040 |
|  | BX537602 |
|  | DA040090 |
|  | DC350705 |
|  | U70451 |
|  | AAB49967 |
|  | U84408 |
|  | AAC50954 |
|  | Q99836 |
|  | NC\_000075 |
|  | NM\_010851 |
|  | NP\_034981 |
|  | AC128702 |
|  | CH466587 |
|  | EDL09224 |
|  | EDL09225 |
|  | EDL09226 |
|  | U89023 |
|  | AAB83958 |
|  | X86457 |
|  | AK090107 |
|  | BAC41096 |
|  | AK150083 |
|  | BAE29292 |
|  | AK151748 |
|  | BAE30658 |
|  | AK152596 |
|  | BAE31345 |
|  | AK152935 |
|  | BAE31609 |
|  | AK167929 |
|  | BAE39933 |
|  | AK186240 |
|  | AK217564 |
|  | BC005591 |
|  | AAH05591 |
|  | BC051075 |
|  | BC058787 |
|  | AAH58787 |
|  | DQ790885 |
|  | ABG78743 |
|  | DQ790886 |
|  | ABG78744 |
|  | KY130516 |
|  | AQQ13080 |
|  | KY130517 |
|  | AQQ13081 |
|  | U84409 |
|  | AAC53013 |
|  | X51397 |
|  | CAA35762 |
|  | P22366 |
|  | NC\_005107 |
|  | XM\_006244087 |
|  | XP\_006244149 |
|  | NM\_198130 |
|  | NP\_937763 |
|  | AC\_000076 |
|  | AABR07073459 |
|  | AAHX01057527 |
|  | CH473954 |
|  | EDL76917 |
|  | AY191270 |
|  | AAO91937 |
|  | BC097266 |
|  | AAH97266 |
|  | Q6Y1S1 |
|  | XP\_006713233 |
|  | XM\_006713170 |
|  | XP\_005265229 |
|  | XM\_005265172 |
|  | AAHY01082338 |
|  | ABBA01024827 |
|  | ACE86707 |
|  | EU831951 |
|  | AC\_000031 |
|  | AC\_000135 |
|  | NC\_018914 |
|  | AABR06057556 |
|  | ACE87392 |
|  | EU832045 |
|  | AMYH02006564 |
|  | NT\_022517 |
|  | NW\_001838877 |
|  | NW\_921651 |
|  | NG\_023225 |
|  | AC\_000046 |
|  | CR619528 |
|  | NT\_039482 |
|  | NW\_001030922 |
|  | Q0PM15 |
|  | Q0PM16 |
|  | Q3TIB3 |
|  | Q3U6W5 |
|  | Q3U7M4 |
|  | Q3UDG9 |
|  | Q8C1X0 |
|  | NW\_001084876 |
|  | NW\_047803 |
|  | Q4QRC0 |
|  | Q53XS7 |
|  | 119584924 |
|  | 119584925 |
|  | 15488923 |
|  | 1763091 |
|  | 1814020 |
|  | 18202671 |
|  | 21757899 |
|  | 30583591 |
|  | 74727050 |
|  | 74728522 |
|  | BAC05209 |
|  | Q8N1J2 |
|  | 110618125 |
|  | 110618127 |
|  | 123782666 |
|  | 123785987 |
|  | 123786657 |
|  | 123787636 |
|  | 123787794 |
|  | 123797080 |
|  | 13542775 |
|  | 148677277 |
|  | 148677278 |
|  | 148677279 |
|  | 1814022 |
|  | 20141515 |
|  | 2459632 |
|  | 26354939 |
|  | 37589152 |
|  | 53294 |
|  | 74198869 |
|  | 74204359 |
|  | 74211897 |
|  | 74220746 |
|  | 74225359 |
|  | 81876106 |
|  | BG072146 |
|  | BG084982 |
|  | 149018276 |
|  | 32732698 |
|  | 67678198 |
|  | 81870890 |
|  | 81918231 |

---

|  |  |
| --- | --- |
| Swiss-Prot ID | MYD88\_HUMAN |
|  | MYD88\_MOUSE |
|  | MYD88\_RAT |
|  | B4DQ60\_HUMAN |
|  | B4DQ72\_HUMAN |
|  | Q3U7M4\_MOUSE |
|  | Q6Y1S1\_RAT |

---

|  |  |
| --- | --- |
| Cell Localization | Cytoplasm |
|  | Nucleus |

---

|  |  |
| --- | --- |
| Ensembl ID | ENSG00000172936 |
|  | ENSP00000497991.1 |
|  | ENST00000650112.1 |
|  | ENSP00000498321.1 |
|  | ENST00000652534.1 |
|  | ENSP00000401399.3 |
|  | ENST00000417037.7 |
|  | ENSP00000499012.1 |
|  | ENST00000651800.1 |
|  | ENSP00000379625.4 |
|  | ENST00000396334.8 |
|  | ENSMUSG00000032508 |
|  | ENSMUSP00000035092.6 |
|  | ENSMUST00000035092.6 |
|  | ENSRNOG00000013634 |
|  | ENSRNOP00000018341.3 |
|  | ENSRNOT00000018341.5 |
|  | ENSP00000379625.3 |
|  | ENST00000495303.5 |
|  | ENST00000424893.5 |
|  | ENSP00000390565.2 |
|  | ENST00000417037.6 |
|  | ENSP00000417848.1 |
|  | ENST00000443433.6 |
|  | ENSP00000389979.1 |
|  | ENST00000396334.7 |
|  | ENSP00000401399.2 |
|  | ENSP00000390565 |
|  | ENSMUST00000035092 |
|  | ENST00000417037 |
|  | ENSP00000417848 |
|  | ENSP00000379625 |
|  | ENSMUSP00000035092 |
|  | ENSRNOT00000018341 |
|  | ENSP00000389979 |
|  | ENST00000495303 |
|  | ENSRNOP00000018341 |
|  | ENSP00000401399 |
|  | ENST00000443433 |
|  | ENST00000424893 |
|  | ENST00000396334 |

---

|  |  |
| --- | --- |
| MGI ID | MGI:108005 |
|  | 108005 |

---

|  |  |
| --- | --- |
| RGD ID | 735043 |

---

|  |  |
| --- | --- |
| Unigene ID | Hs.82116 |
|  | Mm.213003 |
|  | Rn.37341 |

---

|  |  |
| --- | --- |
| Homologene ID | 1849 |

---

|  |  |
| --- | --- |
| IPI ID | IPI00909842 |
|  | IPI00400691 |
|  | IPI00127375 |
|  | IPI00878858 |
|  | IPI00925056 |
|  | IPI00955716 |
|  | IPI00924787 |
|  | IPI00910972 |
|  | IPI00883933 |
|  | IPI00798074 |
|  | IPI00793815 |
|  | IPI00001062 |
|  | IPI00796856 |
|  | IPI00754505 |

---

|  |  |
| --- | --- |
| Homo sapiens Chromosome position | 3p22 |

---

|  |  |
| --- | --- |
| Ensembl Transcript ID | ENST00000396334 |
|  | ENST00000424893 |
|  | ENST00000495303 |
|  | ENST00000443433 |
|  | ENST00000421516 |

---

|  |  |
| --- | --- |
| FunctionalClass | death receptor binding |
|  | protein binding |
|  | signal transducer |
|  | transmembrane receptor |
|  | Toll binding |
|  | receptor binding |

---

|  |  |
| --- | --- |
| Mus musculus Chromosome position | 9 70.0 cM |

---

|  |  |
| --- | --- |
| KEGG pathway | Apoptosis |
|  | Toll-like receptor signaling pathway |

---

|  |  |
| --- | --- |
| Rattus norvegicus Chromosome position | 8q32 |

---

|  |  |
| --- | --- |
| Ensembl Gene ID | LRG\_157 |
|  | ENSG00000172936 |

---
